# Maturity status influences the relative age effect in national top level youth alpine ski racing and soccer

**DOI:** 10.1371/journal.pone.0181810

**Published:** 2017-07-31

**Authors:** Lisa Müller, Christoph Gonaus, Christoph Perner, Erich Müller, Christian Raschner

**Affiliations:** 1 Department of Sport Science, University of Innsbruck, Innsbruck, Tyrol, Austria; 2 Department of Sport Science and Kinesiology, University of Salzburg, Salzburg, Austria; Norwegian University of Science and Technology, NORWAY

## Abstract

Since the relative age effect (RAE) characterizes a problem in all age categories of alpine ski racing and soccer and the fact that, yet, to date the underlying factors have not been well investigated, the aim of the present study was to assess the influence of the biological maturity status on the RAE among youth alpine ski racers (YSR) and soccer players (SP).

In total, 183 male and female YSR selected for national final races and 423 male SP selected for Elite Youth Development Centres were investigated. Additionally, a comparison group of 413 non-athletes was evaluated. The birth months were split into four relative age quarters. The biological maturity status was assessed by the age at peak height velocity (APHV) method; according to the M±SD of the comparison group, the athletes were divided into normal, early and late maturing. Chi^2^-tests indicated a significant RAE among YSR (χ^2^(3,N = 183) = 18.0; p<0.001; ω = 0.31) and SP (χ^2^(3,N = 423) = 33.1; p<0.001; ω = 0.28). In total, only a small number of late maturing athletes were present (0.5–2.3%). Among relatively younger athletes, high percentages of early maturing athletes were found (43.1–43.3%).

The findings indicate that relatively younger and less mature athletes are marginalized or totally excluded in alpine ski racing and soccer. Thus, selection criteria in both sports are effectively based on early biological development and relatively older age, both of which should be considered in future in the talent selection process. In this context, the easy feasible method of assessing the APHV can be used.

## Introduction

The *relative age effect* (RAE) is a well-documented phenomenon in diverse types of sports. A relative age effect exists when the relative age quarter (which corresponds to the birth quarter) distribution of a selected sample of athletes is not as equally distributed among the four quarters as the distribution of the general population is [1]; instead, it shows a skewed distribution with an over-representation of relatively older athletes whose birth months are close to the cut-off date for the competition categories [1,2]. To guarantee fair competition, youth competition categories are classified based on chronological age [2–5]. In this context, January 1 is often used as the cut-off date for each selection year [2,4,6]. As a consequence, athletes competing in the same competition category can differ in age as much as twelve months. These relative age advantages have led to the RAE phenomenon, which was initially documented in Canadian ice hockey [7], and since then, its presence has been proven in many other sports, as well. Two recently published review-articles demonstrated that the RAE in soccer [8] and alpine ski racing [9] is present in all age categories at both national and international levels. Based on these findings, strategies in the talent development systems in these sports should be changed in order to contribute to more fairness because talent in a sport does not depend on the birth month [6]. The existence of a RAE indicates that many relatively younger athletes do not get the chance to reach elite level despite their talents and efforts, they often drop out of sport early and go unnoticed [10–13]. As a consequence, it can be assumed that there is a severe loss of talent due to the existence of the RAE.

Talent identification systems are based on selection biases that confuse maturation for talent. Baker et al. [14] proposed the so-called *maturation hypothesis* to explain RAE in sport. This hypothesis is based on the assumption that the relative age of an athlete is related to his/her cognitive and physical maturation; thus, the favorable selection of relatively older athletes (born early in the selection year) compared to relatively younger athletes (born late in the selection year) is influenced by the maturational differences between them [14]. The short-term consequences are that relatively older and earlier maturing athletes seem to be potentially more “talented” and are favorably selected, whereas relatively younger and less mature athletes are often excluded [15,16]. The combination of a relatively older age and an advanced physical maturation seem to lead to a selection advantage and consequently, to the RAE. This is especially true in sports with high demands on strength and power as evidenced by the selection of youth athletes who were identified as being above average in height and weight compared to age-matched non-athletes [4]. In youth alpine ski racing, Müller et al. [10,11] proved the influence of the biological maturity status on the selection and thus, on the RAE. Athletes selected for national final races were significantly more mature compared to athletes competing only at provincial levels. Additionally, a large number of early maturing athletes were present in the last relative age quarter. This fact showed that relatively younger athletes could counteract their relative age disadvantage by an advanced biological maturity status [10]. In youth soccer, similar results were found [17,18]. Youth soccer players of the four relative age quarters did not significantly differ in the biological maturity status from each other [17]. However, among relatively younger youth soccer players, a high percentage of early maturing athletes was present, whereas among the relatively older players, a large number of late maturing athletes were found [18]. The authors concluded that the relatively older soccer players had an increased likelihood for selection independent of their maturity status, whereas relatively younger athletes often only had a chance of selection if they were early maturing.

Yet, interestingly, no study has been published with regard to biological maturity as a possible influential factor in the most competitive types of sport and across individual sport disciplines and team sport disciplines in the same country. Additionally, it seems important to implement a comparison group of non-athletes of the same age and regions in order to be able to assess whether possible influences of the maturity status on the selection and the RAE are sport specific or generally valid. Therefore, the aim of the present study was to assess the influence of the biological maturity status on the relative age effect in top-level national youth alpine ski racing and soccer, and to implement a comparison group of non-athletes.

## Methods

### Subjects

High level youth ski racers selected for national final races and soccer players from Elite Youth Development Centers (corresponding to the entry point in Austrian youth soccer development programs) participated in this study. The mean age of the subjects was 11.3±0.6 years. In total, 183 national youth ski racers (92 males, 91 females; mean age: 11.6±0.5 years) and 423 male youth soccer players (mean age: 11.1±0.6) were examined. Additionally, a comparison group of 413 non-athletes who were the same age and from the same regions was involved (173 males, 240 females; mean age: 11.5±0.9), who did not perform any types of sport at a specialized high level. Table 1 presents the anthropometric data (means and standard deviations) of the participants, separated by the three groups.

**Table 1:** Anthropometric characteristics separated by soccer players, ski racers and comparison group.

	ski racers		soccer players	comparison group	
Anthropometric characteristic	male	female	male	male	female
	M (±SD)	M (±SD)	M (±SD)	M (±SD)	M (±SD)
Body weight [kg]	41.5 (±6.1)	40.1 (±4.9)	37.0 (±5.7)	41.0 (±10.8)	41.9 (±9.9)
Body height [m]	1.49 (±0.06)	1.50 (±0.06)	1.46 (±0.07)	1.50 (±0.09)	1.51 (±0.09)
Body mass index [kg/m^2^]	18.6 (±1.9)	17.9 (±1.4)	17.3 (±1.7)	18.1 (±3.0)	18.2 (±3.0)
Sitting height [m]	0.77 (±0.03)	0.78 (±0.03)	0.75 (±0.03)	0.77 (±0.04)	0.78 (±0.05)
Age at peak height velocity [yrs]	13.6 (±0.4)	12.0 (±0.4)	13.6 (±0.4)	13.5 (±0.5)	11.9 (±0.4)

M = mean; SD = standard deviation

### Ethics statement

Parents and participants were informed of the study aims before written informed consent was provided. The study was performed according to the Declaration of Helsinki and was approved by the Institutional Review Board of the Department of Sport Science of the University of Innsbruck.

### Measurement and procedures

The present study is an observational research study. The birth dates of all participants were collected and were then categorized into four relative age quarters according to their birth months. January 1 is used as the cut-off date for the competition categories in alpine ski racing and soccer; thus, the months were split into quarters to calculate the relative age quarters as follows: January to March were categorized as relative age quarter 1 (Q1); April to June as quarter 2 (Q2); July to September as quarter 3 (Q3), and October to December as quarter 4 (Q4).

The biological maturity status was investigated using the non-invasive method of calculating the *age at peak height velocity* (APHV) [19]. The gender-specific prediction equations include the following anthropometric parameters, which were assessed by two independent technicians according to previously described procedures [20]: body height (0.1 cm, Seca Portable Stadiometer, Hamburg, Germany), sitting height (0.1 cm, Seca Portable Stadiometer, Hamburg, Germany; sitting height table) and body mass (0.1 kg, Seca, Hamburg, Germany). The inter-rater reliability was perfect (ICC = 1.0; p<0.001) for body height, body weight and sitting height. The leg length as difference between body height and sitting height and actual chronological age at the time of measurement were calculated and included in the equations. Based on this, the *maturity offset* (MO), the time before or after individual peak height velocity (PHV), could then be assessed in order to calculate the predicted APHV as difference between chronological age and MO. Validity of this method was previously proven among youth ski racers of the same age as those who participated in the present study [21]. As suggested [22], the participants were then divided into three groups of maturity (late, normal and early maturing) based on the mean (M) ± standard deviation (SD) of the APHV of the comparison group of age-matched pupils (separated by gender). An athlete was classified as normal maturing if his/her APHV was within M ± SD; he/she was early maturing if the APHV was less than M–SD, and late maturing if it was higher than M + SD. Validity of this classification was previously shown among youth ski racers and age-matched pupils [21].

### Statistical analyses

To assess the difference between the observed and the expected relative age quarter distributions, chi^2^-tests (χ^2^) were used for the two groups of athletes (ski racers and soccer players). The relative age quarter distribution of the comparison group of non-athletes, which corresponded to a nearly even distribution among the four quarters (nearly 25% in each quarter), was used as the expected distribution for these analyses. The effect size ω was calculated for the χ^2^-tests [22]. Odds ratio (OR) and 95% confidence intervals (95% CI) were calculated [4].

Kolmogorov-Smirnov tests were used to assess the normal distribution of the APHV (separated by gender, group of athletes and single relative age quarters). To assess differences in the APHV between the four relative age quarters (separated by gender), univariate analyses of variance were used (dependent variable: APHV; independent variable: relative age quarter). The variance homogeneity was assessed with Levene-Test and for post-hoc-tests, those of Scheffé were used. χ^2^-tests were used to evaluate the difference between the expected (normal) distribution of early, normal and late maturing athletes among each relative age quarter and the observed distribution.

The level of significance was set at p<0.05. All of the calculations were performed using IBM SPSS 23.0 (IBM Corporation, Armonk, NY, USA); the effect size was assessed using G*Power 3.1.9.2 (University of Düsseldorf, Germany).

## Results

### Relative age effect

**Fig 1:**
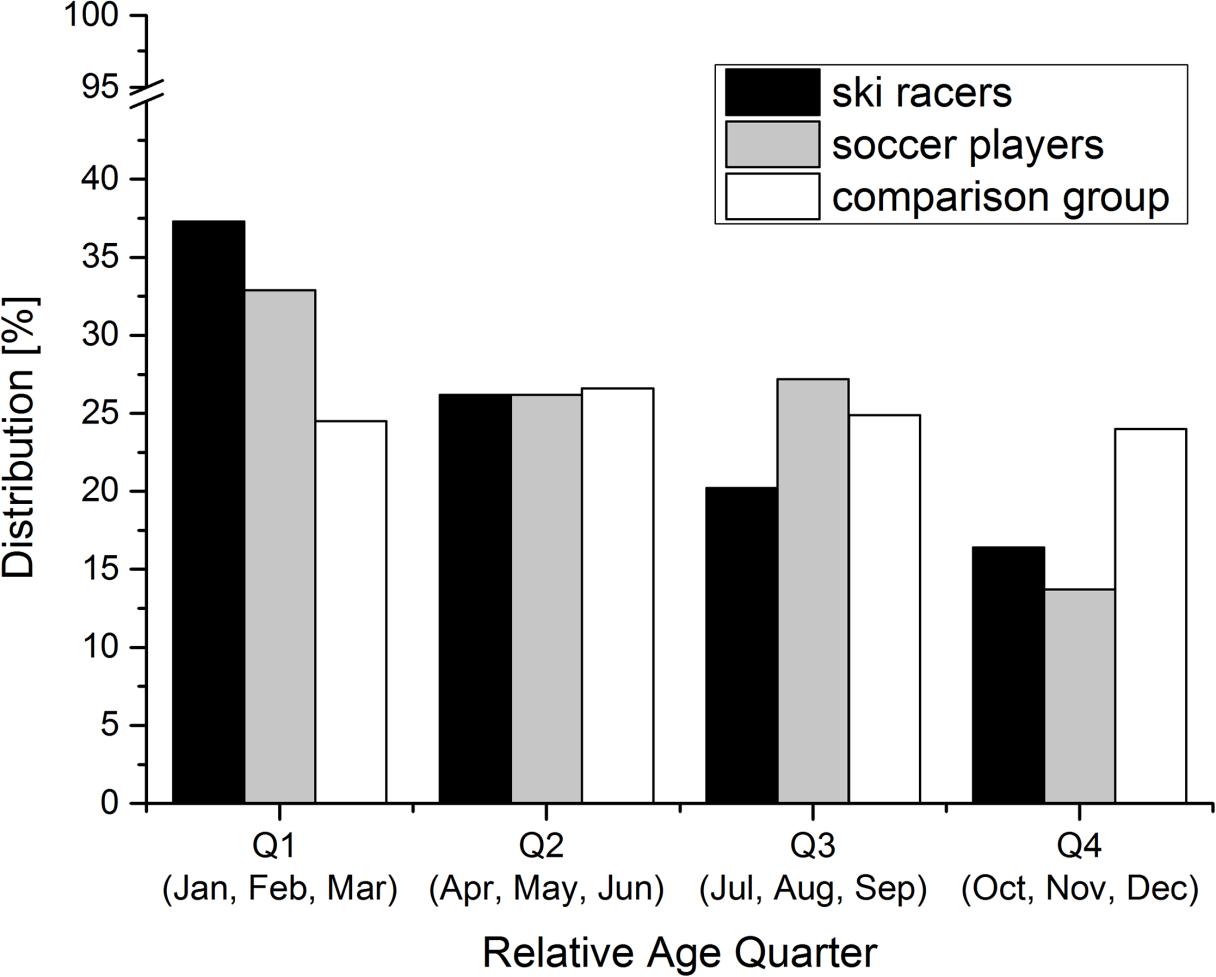
Relative age quarter distribution of ski racers, soccer players and comparison group.

A highly significant RAE was found among the ski racers and the soccer players. The relative age quarter distribution of the ski racers significantly differed from the distribution of the comparison group with an over-representation of athletes of Q1 (χ^2^(3, N = 183) = 18.0; p<0.001; ω = 0.31). The distribution of the soccer players significantly differed from the distribution of the comparison group, as well (χ^2^(3, N = 423) = 33.1; p<0.001; ω = 0.28). The relative age quarter distributions of the ski racers, soccer players and the comparison group are presented in Fig 1. The descriptive OR and the corresponding χ^2^ for each quarter of the ski racers and the soccer players are presented in Table 2. The OR calculations revealed significant differences in the ski racers between Q1 and Q3 and Q4; a tendency was present also in the comparison of Q1 and Q2 (p = 0.063). Among the soccer players significant differences were found between Q1 and Q4. The likelihood of selection for national final races (ski racers) and Elite Youth Development Centers (soccer players) of an athlete of Q1 was 5.1 times higher for a ski racer and 5.7 times higher for a soccer player compared to athletes of the last relative age quarter. Among the ski racers the likelihood of an athlete of Q1 was 3.4 times higher than for an athlete of Q3.

**Table 2:** Descriptive OR across all relative age quarters according to ski racers and soccer players.

Sample			Q1:Q2	Q1:Q3	Q1:Q4
Ski racers	Total (n = 183)	Chi^2^	3.45	9.15	14.74
		*p* value	0.063	0.002	<0.001
		*OR* [95% CI]	**2.01 (1.19–3.38)**	**3.38 (1.92–5.95)**	**5.14 (2.80–9.43)**
Soccer players	Total (n = 423)	Chi^2^	3.14	2.27	33.31
		*p* value	0.077	0.132	<0.001
		*OR* [95% CI]	**1.57 (1.10–2.23)**	**1.46 (1.03–2.07)**	**5.74 (3.72–8.86)**

*OR* = odds.ratio; *CI* = Confidence Interval; Q1-4 = relative age quarter 1–4. **Bolded values indicate significance of odds ratio (if 95% does not include 1).**

### Influence of biological maturity status

The analyses of variance showed that the male and female ski racers of the four relative age quarters did not significantly differ from each other in the APHV. However, the soccer players of the four relative age quarters did significantly differ in the APHV from each other (F(3, 423) = 10.86; p<0.001). Post-hoc-tests showed that athletes of Q3 (mean APHV = 13.56±0.34 years) and Q4 (13.48±0.32 years) significantly differed from athletes of Q1 (13.75±0.38 years) and Q2 (13.70±0.36 years).

Most of the ski racers (78.1%) were normal maturing; 21.4% were early maturing and 0.5% were late maturing. Among the soccer players, 73.8% were normal maturing, 23.9% were early maturing and 2.3% were late maturing. A significant difference was present between the expected normal distribution of early, normal and late maturing athletes and the observed distributions of the ski racers (χ^2^(3, N = 183) = 32.53; p<0.001; ω = 0.42) and the soccer players (χ^2^(3, N = 423) = 78.66; p<0.001; ω = 0.43). The distributions of normal, early and late maturing ski racers and soccer players are presented in Table 3, separated by relative age quarter.

**Table 3:** Percentages of normal, early and late maturing athletes separated by ski racers and soccer players.

		Relative age quarter			
		Q1	Q2	Q3	Q4
		[%]	[%]	[%]	[%]
Ski racers	normal	86.8	75.0	83.8	56.7
	early	13.2	22.9	16.2	43.3
	late	0	2.1	0	0
Soccer players	normal	75.5	82.9	71.3	56.9
	early	19.5	14.5	28.7	43.1
	late	5.0	2.6	0	0
Comparison group	normal	70.3	74.6	74.8	67.6
	early	12.9	12.7	15.5	17.2
	late	16.8	12.7	9.7	15.2

Q1-4 = relative age quarter 1–4

## Discussion

The present study is the first study that assessed the influence of the biological maturity status on the relative age effect among youth athletes of the two most competitive types of sport in Austria, alpine ski racing and soccer. Additionally, a comparison group of non-athletes was included to allow direct comparisons with age-matched pupils of the same regions. In both types of sport, a highly significant RAE was found. Additionally, soccer players of the last two relative age quarters were significantly more mature than the relatively older players. The distribution of normal, early and late maturing athletes significantly differed from the expected normal distribution in both groups of athletes; hardly any athletes were late maturing. Moreover, in both groups, high percentages of early maturing athletes were present among athletes of the last relative age quarter. Thus, the biological maturity status strongly influences the RAE both in youth alpine ski racing and in soccer.

It was not surprising that in the present study a significant RAE was found among youth ski racers and soccer players with an over-representation of athletes born early in the selection year, particularly since the observations of previous studies [8,9] supported these findings. Both groups of athletes represent the most “talented” 10 to 12 year old athletes who were selected for national final races or national Youth Development Centers. The ski racers had a larger effect size (ω = 0.31) compared with the soccer players (ω = 0.28). However, the effect size of the ski racers is comparable to the study of Müller et al. [11]. The OR calculations clearly demonstrated the selection bias of identifying the most talented youth athletes. The likelihood of the selection of ski racers of Q1 significantly differed from Q3 and Q4; the difference was greatest between Q1 and Q4 because relatively older athletes had a 5.1 (CI: 2.80–9.43) times higher likelihood of selection for the national final races compared with relatively younger athletes. In the study of Müller et al. [11], the likelihood was slightly smaller with 3.7 (CI: 2.05–6.63). In soccer, the likelihood of selection significantly differed only between Q1 and Q4; however, the likelihood of selection for the Youth Development Centers was as much as 5.7 (CI: 3.72–8.86) times higher for an athlete of the first compared with an athlete of the last relative age quarter. The magnitude of the selection bias was much greater than in a comparable study [23], in which OR of 2.7 (CI: 1.7–4.3) was found for under 11 and of 2.1 (CI: 1.4–3.2) for under 12 year old Scottish soccer players. The OR of soccer players of Q1 and Q3 was relatively small (OR 1.46; CI: 1.03–2.07), which was surprising and might be explained by the Austrian school classification system, in which the cut-off date is August 31^st^. As a consequence, pupils of the third relative age quarter are the “oldest” in the school class, and probably this might have contributed to the small OR between Q1 and Q3. The greater differences between Q1 and Q4 in the present study may be due to the high selection pressure during the selection process in these two types of sport in Austria, which is seen as a precondition for RAE [1]. The talent development systems in an individual sport like alpine ski racing and in a team sport like soccer are clearly biased and discriminate against relatively younger athletes. In order to be able to change strategies in the talent development systems, the role of the biological maturity status as possible influential factor in the talent selection process was evaluated.

More mature athletes are often favorably selected in team sports like soccer [18,24] and basketball [25], as well as in individual sports like alpine ski racing [11]. In both sports, an advanced biological maturity status is associated with sport specific performance advantages [26]. Thus, it can be assumed that more mature athletes have selection advantages, which could be confirmed by the findings of the present study. By dividing the athletes in three groups of maturity status based on the M±SD of the comparison group of non-athletes, it was observed that in both groups, hardly any late maturing athletes were present (ski racers: 0.5%; soccer players: 2.3%). Additionally, a significant difference between the expected normal distribution and the observed distribution of normal, early and late maturing ski racers (ω = 0.42) and soccer players (ω = 0.43) was found. These findings are in line with the results of other studies in youth ski racing [11] and soccer [24,27], in which a predominance of early maturers was found among selected athletes. Additionally, Johnson et al. [28] showed that maturation status had an even 10-fold stronger influence on selection in elite youth soccer than the relative age.

The most frequent explanation for the RAE phenomenon is the maturation-selection hypothesis [29]. Additionally, previous studies have reported that relatively younger athletes can counteract their relative age disadvantage if they are endowed with an advanced biological maturity status; however, relatively older athletes have an increased likelihood of selection independent of their maturity status [18]. In the present study, a significant difference in APHV between the four relative age quarters only was found among the soccer players; the relatively younger athletes will reach their individual peak growth spurt at a significantly younger age, which indicates that they were more mature compared to athletes of Q1 and Q2. In this context, in literature contrasting results can be found: Lovell et al. [30] revealed similar findings for the under-10 and under-18 soccer players, whereas no differences were assessed for the under-12, under-14 and under-16 players. Additionally, Gil et al. [17] did not find any significant differences in APHV between the quarters, which is comparable to alpine ski racing [11], and again in line with the results of the present study among the ski racers.

When comparing the classification of normal, early and late maturing athletes separated by relative age quarter, a difference between ski racing and soccer can be seen in the literature. In national youth ski racing, Müller et al. [11] reported that in all four relative age quarters, only a small number of late maturers were present (0–8.3%) and among the relatively youngest, high percentages of early maturing athletes were found (females: 34%; males: 41.7%). In contrast, in soccer, a high percentage of late maturing athletes was present (U13: 41.3%) among the relatively oldest players, whereas among the relatively youngest players, 33.3% were early maturing and 27.8% late maturing. A possible explanation could be that an advanced biological maturity status is more advantageous in the selection process in ski racing because more mature athletes benefit from early recognition from coaches and talent scouts more than in soccer. Additionally, in ski racing, selection processes are primarily based on race results, and in this context, more mature athletes clearly have advantages, although this selection strategy is short-sighted because it is based on advantages that are no longer relevant after adolescence [25]. However, in soccer, the selection criteria are based on diverse factors [31], one of which is the playing technique, and it can be assumed that not only early maturers, but also less mature players could benefit from a well-developed technique. These differences between ski racing and soccer were not apparent in the present study. A trend can be seen among the late maturing soccer players because despite the fact that hardly any athletes were late maturing (5% of the athletes of Q1 and 2.6% of Q2 were late maturing), no late maturers were born in Q3 and Q4. Nonetheless, the small percentages have to be considered and it can only be interpreted as a trend, which would be in line with Deprez et al. [18]. A clear trend was present among the early maturing soccer players: nearly half of the athletes of Q4 (43.1%) and nearly a third of the athletes of Q3 (28.7%) were early maturing. Similar results were present among the ski racers, of whom 43.3% of the relatively youngest were early maturing. Thus, it can be concluded that relatively younger youth soccer players and youth ski racers, for the most part, only have a chance of selection if they are early maturing. In contrast to Deprez et al. [18], the likelihood of selection of the relatively oldest athletes is not independent of the maturity status, but it can be interpreted that at least they do not have to be early maturing to get selected. As described by Torres-Unda et al. [25] in basketball, relatively younger and less mature athletes may be denied access to professional training; consequently, they do not have the same opportunities for reaching their full potential.

## Limitations

In the present study, the sample sizes of soccer players and ski racers were different because of the fact that at this high level of youth sport specialization, more soccer players were available and the ski racers selected for national final races were already investigated over four years. However, this concern has to be mentioned a limitation of the study. Additionally, no female soccer players were included in the study because the selection pressure in female soccer in Austria is not that high and at a comparable level only a small number of female soccer players would be available. Additionally, no relative age effect exists in female soccer in Austria. Nevertheless, this concern has to be considered as limitation of the study. Moreover, the analyses with respect to the influence of biological maturity on the relative age effect were performed combined for male and female ski racers because otherwise the sample sizes would be relatively small. However, among both male and female ski racers a significant relative age effect and a significant difference in the distribution of normal, early and late maturing athletes from the expected normal distribution was observed. Among the female athletes, no late maturing ski racers were present (17.6% early maturing), only 1.1% of the male athletes were late maturing. Because the advantages of an advanced maturation are high for both male and female ski racers, the analyses were performed for both genders combined. However, this has to be considered as limitation of the study.

Additionally, the classification of normal, early and late maturing athletes might lack specificity. However, for this reason the soccer players and ski racers were categorized in the three groups based on the mean ± SD of the comparison group of the same regions in order to have representative categories. Nevertheless, the small differences in APHV could exist between athletes categorized as normal or late maturing. Therefore, this should be seen as limitation of the study, particularly because of the small standard deviations of the comparison group. However, this categorization was previously compared with the classification based on X-rays of the left wrist [21], and is often used in studies among youth athletes.

## Conclusion

The RAE represents a severe problem in alpine ski racing and soccer because many talented athletes get lost and cannot fulfil their potential [8,9]. Thus, it is necessary to assess the influential factors of the RAE in each specific sport [29]. Gorski et al. [13] reported that some suggestions to reduce the RAE, such as a rotating cut-off date and new competition group classifications based on maturation, cannot be implemented and that coaches and federations should be aware of the RAE in the talent selection process. The present study was the first study to clearly demonstrate that the biological maturity status strongly influences the RAE in alpine ski racing and soccer, and that less mature and relatively younger athletes nearly do not have the chance for selection. Based on these findings, in the future, the biological maturity status should be assessed in the talent selection process to not exclude less mature and relatively younger athletes. In this context, the easy feasible tool of assessing the APHV can be used [21].

Selection criteria in alpine ski racing and soccer are effectively based on early biological development and a relatively older age. Relatively younger and late maturing athletes seem to be marginalized or totally excluded; their careers seem to be suppressed by the relative age disadvantage associated with inexperience [25]. In the future, the relative age and biological maturity status should be considered in the talent selection process in alpine ski racing and soccer in order to give relatively younger and late maturing athletes more time to fulfil their potential and to not exclude them in advance. Additionally, in the education program of coaches in alpine ski racing, a greater awareness of these problems should be created; race results should not be overvalued.
